# Axin-1 Regulates Meiotic Spindle Organization in Mouse Oocytes

**DOI:** 10.1371/journal.pone.0157197

**Published:** 2016-06-10

**Authors:** Xiao-Qin He, Yue-Qiang Song, Rui Liu, Yu Liu, Fei Zhang, Zhen Zhang, Yu-Ting Shen, Lin Xu, Ming-Huang Chen, Ya-Long Wang, Bai-Hui Xu, Xiang-Jun Yang, Hai-Long Wang

**Affiliations:** 1 Organ Transplantation Institute, Medical College, Xiamen University, Xiamen City, Fujian Province, P. R. China; 2 Department of Gynaecology and Obstetrics, Zhongshan Hospital, Xiamen University, Xiamen City, Fujian Province, P. R. China; 3 New England Fertility Institute, Stamford, CT, United States of America; 4 Xiamen Institute for Food and Drug Quality Control, Xiamen City, Fujian Province, P. R. China; 5 Department of Obstetrics and Gynecology, Shanghai First Maternity and Infant Hospital, Tongji University School of Medicine, Shanghai City, P. R. China; 6 The Fifth Hospital of Wuhan, Wuhan City, Hubei Province, P. R. China; Institute of Zoology, Chinese Academy of Sciences, CHINA

## Abstract

Axin-1, a negative regulator of Wnt signaling, is a versatile scaffold protein involved in centrosome separation and spindle assembly in mitosis, but its function in mammalian oogenesis remains unknown. Here we examined the localization and function of Axin-1 during meiotic maturation in mouse oocytes. Immunofluorescence analysis showed that Axin-1 was localized around the spindle. Knockdown of the Axin1 gene by microinjection of specific short interfering (si)RNA into the oocyte cytoplasm resulted in severely defective spindles, misaligned chromosomes, failure of first polar body (PB1) extrusion, and impaired pronuclear formation. However, supplementing the culture medium with the Wnt pathway activator LiCl improved spindle morphology and pronuclear formation. Downregulation of Axin1 gene expression also impaired the spindle pole localization of γ-tubulin/Nek9 and resulted in retention of the spindle assembly checkpoint protein BubR1 at kinetochores after 8.5 h of culture. Our results suggest that Axin-1 is critical for spindle organization and cell cycle progression during meiotic maturation in mouse oocytes.

## Introduction

Mammalian oocyte maturation is a multi-stage, accurately orchestrated and orderly process [1, 2]. Germinal vesicle breakdown (GVBD) indicates the beginning of oocyte maturation. After that, microtubules assemble to form a bipolar structure around the chromosomes during prometaphase I (Pro MI). Chromosomes migrate to the spindle equator at metaphase I (MI). Subsequently, the spindle migrates to the cortex and the oocyte emits the first polar body (PB1), followed by the formation of the metaphase II (MII) spindle beneath the plasma membrane [3]. In this process, two critical events are Pro MI arrest and progression through the first meiotic division [4]. The correct formation and organization of the meiotic spindle is essential for accurate chromosome organization, congression, and segregation in meiotic maturation. Meiotic spindle formation in mouse oocytes relies on microtubule organizing centers (MTOCs) that lack centrioles [5, 6], and these mainly consist of centrosomal proteins, such as γ-tubulin, p38α, and Nek9 [7, 8].

Wnt signaling pathways regulate a number of biological processes such as cell proliferation, motility and oncogenesis [9]. Various components of these pathways are implicated in chromosomal instability and mitosis [10–13]. Axin, a key constituent of Wnt signaling, is a scaffold protein implicated in multiple signaling pathways including mitogen-activated protein kinase activation, and p53 signaling in responses to DNA damage [14–16]. Axin genes include Axin1 and its homologue Axin2. Compared to the multi-function of Axin2 in cell proliferation, cytometaplasia, migration, apoptosis and other cell functions, the Axin-1 protein is considered to localized to the centrosome and forms a complex with γ-tubulin. Depletion of Axin1 gene expression was shown to affect the localization of γ-tubulin and centrosomal microtubule nucleation [17]. In addition, Axin-1 protein has been shown to localize at the centrosome and along mitotic spindles, and to regulate the distribution of polo-like kinase 1 and glycogen synthase kinase 3 beta. These interactions affect mitotic processes, such as cytokinesis in the presence of the Aurora kinase inhibitor [18]. In addition, phosphorylation of Axin-1 by PLK1 plays a crucial role in controlling its association with γ-tubulin, centrosome formation, and segregation[19].

Although Axin-1 plays key roles in mitosis, whether it participates in meiosis remains unknown. Here, we used immunofluorescence staining to examine its localization, and used specific short interfering (si)RNA sequences to examine its functions in mouse oocyte meiosis.

## Materials and Methods

### Ethics statement

Mice care and use were conducted in strict accordance with the recommendations in the Guide for the Care and Use of Laboratory Animals of the National Institutes of Health. The protocol was approved by Animal Studies Committee of Xiamen University, China (approval ID: XMUMC 2011-10-08). ICR mice were housed and bred at temperature-controlled room, received the standard murine chow diet, and kept on a cycle of 12 h light and 12 h dark, with the darkness starting from 19:00. The mice were killed by cervical dislocation. All efforts were made to minimize suffering and the only procedures performed on the dead animals were getting ovaries and the collection of oocytes from the ovaries.

### Antibodies and chemicals

A rabbit polyclonal anti-Axin-1 antibody was purchased from Millipore (Billerica, MA, USA). Sheep polyclonal anti-BubR1 and rabbit polyclonal anti-Nek9 antibodies were from Abcam (Boston, USA). Mouse monoclonal anti-γ-tubulin antibody, anti-α-tubulin FITC-tagged antibody, and TRITC-tagged anti-phalloidin were obtained from Sigma-Aldrich (St Louis, USA). Alexa Fluor 546-conjugated donkey anti-rabbit and donkey anti-sheep antibodies were purchased from Life Technologies (Carlsbad, USA). An Alexa Fluor 546-conjugated donkey anti-mouse antibody was purchased from Invitrogen Life Technologies (Carlsbad, USA). Control rabbit IgG was purchased from Santa Cruz Biochemicals (Dallas, USA). Mounting medium containing DAPI was purchased from Vector Laboratories (Burlingame, USA) and milrinone was purchased from Cayman Chemical Co. (Ann Arbor, USA).

### Oocyte collection and culture

Germinal vesicle (GV)-intact oocytes were collected from the ovaries of 6- to 8-week-old female ICR mice in M2 medium (Sigma-Aldrich, St Louis, USA) supplemented with or without 2.5 μM milrinone. The oocytes were washed thoroughly and cultured in M2 medium under sterile liquid paraffin oil at 37°C in an atmosphere of 5% CO_2_ in humidified air. Oocytes were collected for treatment or examination at the indicated times during culture.

### Real-time quantitative polymerase chain reaction (PCR) analysis

Analysis of Axin1 gene mRNA expression was measured by real-time quantitative PCR and the ΔΔC_T_ method. We used a Dynabead mRNA DIRECT kit (Life Technologies AS, Oslo, Norway) to extract the total RNA from 70 oocytes. First strand cDNA was generated using a cDNA synthesis kit (Toyobo, Tokyo, Japan), using Oligo(dT) 12–18 nucleotide primers (Takara Bio Inc., Tokyo, Japan). A cDNA fragment of Axin1 was amplified using the following primers: forward, CAC CCA GAA GCT GCT ATT GGA GA, reverse, CCA GGG CAT AGC CAG AGT TGA. We used SYBR Green real-time PCR Master Mix kits (Life Technologies, Carlsbad, USA) with a Step One real-time PCR system (Applied Biosystems, Foster City, USA) under the following conditions: 50°C for 2 min and 95°C for 2 min; followed by 40 cycles of 95°C for 15 s and 60°C for 1 min.

### Axin1 siRNA microinjection

Aliquots of ~5–10 pL of 50 μM Axin1 siRNA (GenePharma, Shanghai, P. R. China) (5′-GGG AGC UAC AGA UAC UAC UTT-3′; 5′-AGU AGU AUC UGU AGC UCC CTT-3′) were microinjected into the cytoplasm of fully grown GV stage oocytes, using a Nikon Diaphot ECLIPSE TE300 inverted microscope (Nikon UK Ltd., Kingston upon Thames, UK) equipped with a Narishige MM0-202N hydraulic three-dimensional micromanipulator (Narishige Inc., Sea Cliff, USA). After microinjection, the oocytes were cultured for 24 h in M2 medium containing 2.5 μM milrinone, and then given three 2-min washes in fresh M2 medium. The oocytes were the transferred to M2 medium for 8.5 h or 12 h with 2.5 μM LiCl under sterile paraffin oil at 37°C under 5% CO_2_ in humidified air. Control oocytes were microinjected with 5–10 pL of negative control siRNA. The spindle phenotypes and chromosome alignment were examined using confocal microscopy. Polar body extrusion was observed using a stereomicroscope.

### Western blotting

Groups of 200 mouse oocytes were collected in sodium dodecyl sulfate (SDS) sample buffer and heated for 5 min at 100°C. The proteins were separated by SDS–polyacrylamide gel electrophoresis (PAGE), transferred electrically to polyvinylidene fluoride membranes, and then blocked in TBST containing 5% skimmed milk for 1 h, followed by incubation overnight at 4°C with a rabbit polyclonal anti-Axin-1 antibody (1:800, Millipore) and a mouse monoclonal anti-β-actin antibody (1:10000, Abcam, Cambridge, UK). After three 10-min washes in TBST, the membranes were incubated for 1 h at 37°C with peroxidase-conjugated rabbit anti-mouse IgG (1:1000, Zhong Shan Jin Qiao Co., Beijing, P. R. China) and peroxidase-conjugated mouse anti-rabbit IgG (1:1000, Zhong Shan Jin Qiao Co., P. R. China). Finally, the membranes were processed using SuperSignal West Femto maximum sensitivity substrates.

### Immunofluorescence and confocal microscopy

The protocol was essentially as described [20–22]. For single immunostaining of Axin-1, α-tubulin, BubR1, or control rabbit IgG, oocytes were fixed in 4% paraformaldehyde in phosphate-buffered saline (PBS) for 30 min at room temperature. After being permeabilized with 0.5% Triton X-100 at room temperature for 30 min, oocytes were blocked in PBS supplemented with 1% bovine serum albumen (BSA) for 1 h and incubated overnight at 4°C with 1:100 diluted rabbit anti-Axin-1 antibody, 1:200 mouse anti-α-tubulin-FITC antibody, 1:25 sheep polyclonal anti-BubR1 antibody or 1:50 control rabbit IgG; all in PBS. After three 5-min washes in PBS containing 0.1% Tween 20 and 0.01% Triton X-100, the oocytes were labeled with 1:250 donkey anti-sheep IgG antibodies conjugated with Alexa Fluor 546 (to stain BubR1), or 1:500 donkey Anti-Rabbit IgG antibodies conjugated with Alexa Fluor 546 (to stain Axin-1) for 1 h at room temperature (this step was omitted for immunostaining of α-tubulin).

For double immunostaining of γ- and α-tubulin, after being fixed, permeabilized and blocked as above, oocytes were incubated overnight at 4°C with a 1:50 mouse monoclonal anti-γ-tubulin antibody in PBS. After three 5-min washes in washing buffer (PBS containing 0.1% Tween 20 and 0.01% Triton X-100), the oocytes were labeled with 1:350 donkey anti-mouse IgG antibodies conjugated with Alexa Fluor 546 for 1 h. After γ-tubulin immunostaining, the oocytes were again blocked in 1% PBS with 1% BSA for 1 h at room temperature, followed by immunostaining with 1:200 mouse anti-α-tubulin–FITC antibody for another 1 h.

For double immunostaining of Nek9 and α-tubulin, after being fixed, permeabilized, and blocked as above, oocytes were incubated overnight at 4°C with a 1:40 mouse monoclonal anti-Nek9 antibody. After three 5-min washes in washing buffer as above, the oocytes were immunolabeled with 1:400 donkey anti-rabbit IgG antibodies conjugated with Alexa Fluor 546 in PBS for 1 h. The oocytes were again blocked in 1% PBS with 1% BSA for 1 h at room temperature, followed by immunostaining with 1:200 mouse anti-α-tubulin–FITC antibody in PBS for 1 h. After three 5-min washes in PBS containing 0.1% Tween 20 and 0.01% Triton X-100, the oocytes were mounted on glass slides with mounting medium containing DAPI for staining DNA. Finally, the oocytes were examined using an FV1000 confocal laser-scanning microscope (Olympus, Tokyo, Japan).

### Parthenogenetic activation

The activation medium was Ca^2+^- free CZB medium supplemented with 10 mM SrCl_2_ and 5 μg/ml cytochalasin B. All samples were activated simultaneously. Oocytes were washed three times in activation solution and transferred to activation medium for 6 h, then examined under a stereomicroscope to observe pronuclear formation.

### Statistical analysis

At least three replicates were conducted for each treatment. Data (mean ± standard error of the mean, SEM) were analyzed using analysis of variance using Graph-Pad Prism software (v. 5; La Jolla, USA) followed by Student–Newman–Keuls test, and P < 0.05 was considered significant. Different superscripts in figures and tables indicate statistically significant differences and the numbers of oocytes are labeled in parentheses as (n).

## Results

### Subcellular localization of Axin-1 during meiotic maturation in mouse oocytes

**Fig 1:**
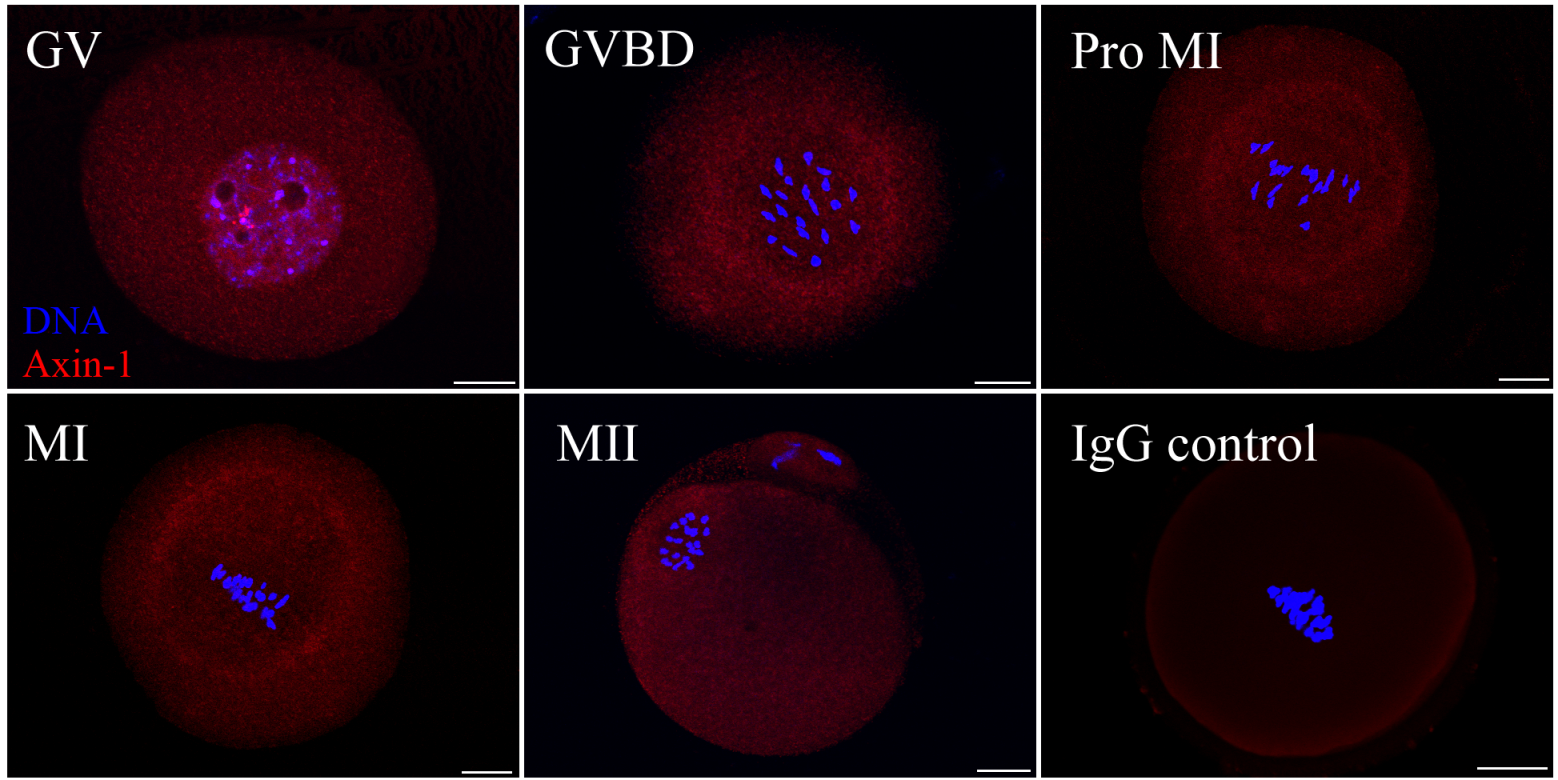
Subcellular localization of Axin-1 during meiotic maturation in mouse oocytes. Oocytes at various stages were stained with an antibody against Axin-1 (red) and each was counterstained with DAPI to visualize DNA (blue). Key: GV, oocytes at germinal vesicle stage; GVBD, oocytes at germinal vesicle breakdown; Pro MI, oocytes at the first prometaphase stage; MI, oocytes at the first metaphase stage; MII, oocytes at the second metaphase stage. Oocytes in the negative control group were incubated with rabbit IgG. Scale bar = 20 μm.

As shown in **Fig 1**, Axin-1 was distributed throughout the cytoplasm and seemingly concentrated around the germinal vesicle at the GV stage. After GVBD, an accumulation of dotted structures of Axin-1 appeared in the central part of the oocyte. When oocytes came to prometaphase I, Axin-1 was localized around the spindle, as the chromosomes began to arrange themselves. And by MI, when chromosomes were regularly aligned at the equatorial plate, Axin-1 was found around the spindles. After the formation of the second meiotic spindle at MII, Axin-1 presented the same localization, was found around the spindles. The negative IgG control showed no signals.

### Knockdown of Axin1 gene expression led to abnormal meiotic spindles and misaligned chromosomes

**Fig 2:**
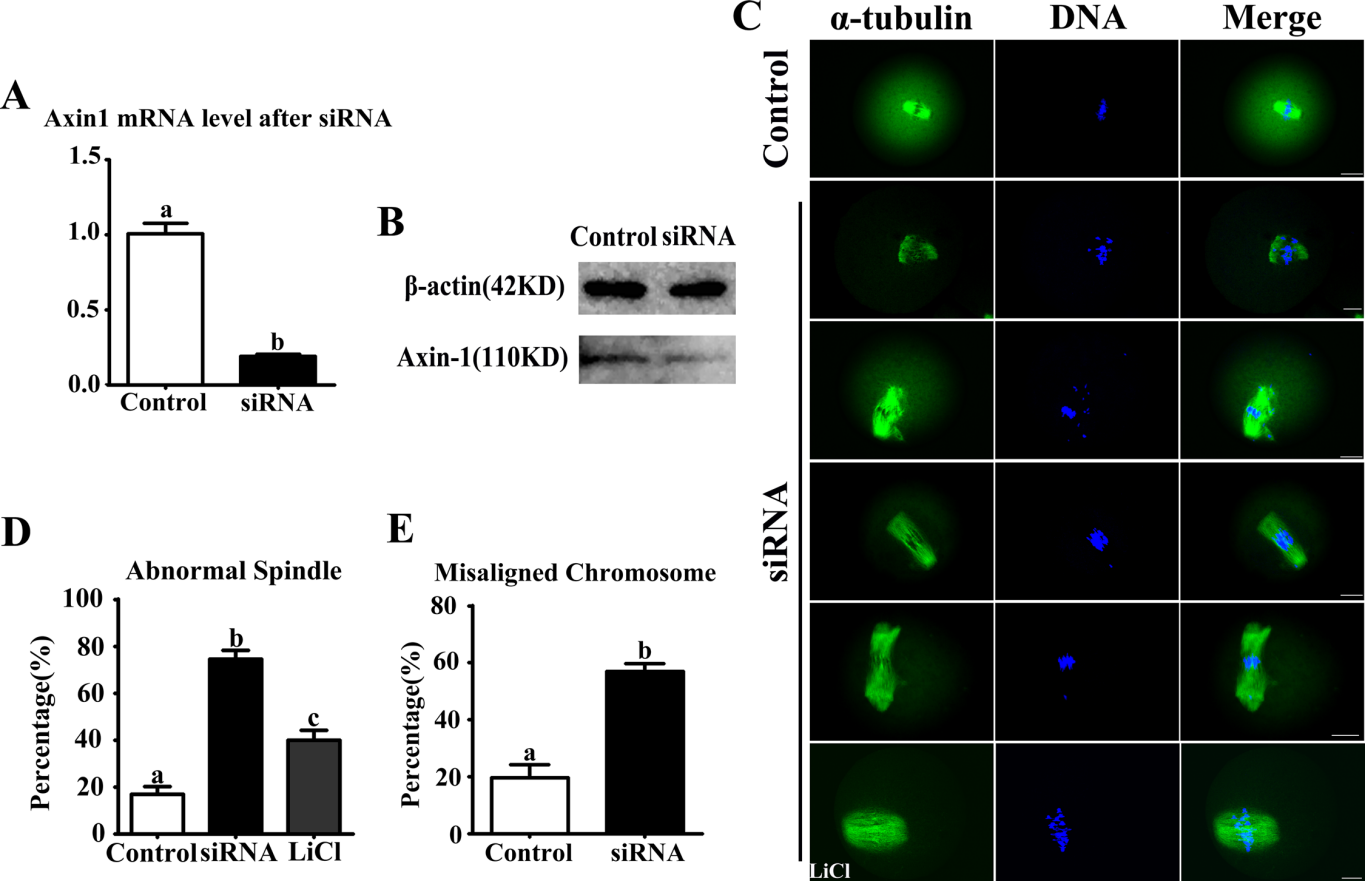
Disruption of Axin-1 function adversely affected spindle assembly and chromosome alignment in MI oocytes. (A) Axin1 mRNA level after siRNA microinjection. (B) Axin-1 protein expression level after siRNA microinjection. (C) Oocytes microinjected with Axin1 or control siRNAs were collected after 8.5 h of culture in fresh M2 medium. In the Axin1-specific siRNA-injected group, the oocytes exhibited various morphologically defective spindles and misaligned chromosomes. However, when supplemented with LiCl, the spindles morphology were rescued. (D) Percentages of oocytes with abnormal spindles in the Axin1 siRNA-injected group (n = 92) and control group (n = 94) and LiCl group (n = 89). Data are presented as the mean ± SEM. Different superscripts indicate significant differences (P < 0.05). (E) Percentages of oocytes with misaligned chromosomes in the Axin1 siRNA-injected group (n = 92) and control group (n = 90). Data are presented as the mean ± SEM. Superscripts indicate statistically significant differences (P < 0.05).

Axin1 siRNA microinjection successfully depressed the mRNA level (19.0 ± 0.1% vs. control at 100.9 ± 1.0%, n = 210; P < 0.05) (**Fig 2A**). We also detected the expression of Axin-1 by western blotting (**Fig 2B**). After siRNA microinjection, the oocytes were collected after 8.5 h culture and used for further immunostaining. In the Axin1 siRNA-microinjection group, oocytes exhibited different kinds of morphologically defective spindles, including no poles, single poles, or multiple poles, along with elongated and deformed spindles (**Fig 2C**). However, normal spindles were present in the LiCl treatment group. The rate of abnormal spindle formation in the Axin1 siRNA-injected group was significantly higher than in the control group (74.6 ± 3.6%, n = 92 vs. 17.1 ± 3.1%, n = 94 in controls; P < 0.05) (**Fig 2D**). In addition, the incidence of misaligned chromosomes in the Axin1 siRNA-injected group was up to 56.9 ± 2.8%, much higher than in the control group (19.8 ± 4.4%; P < 0.05) (**Fig 2E**).

### Knockdown of Axin1 gene expression caused disruption of γ-tubulin and Nek9 localizations

**Fig 3:**
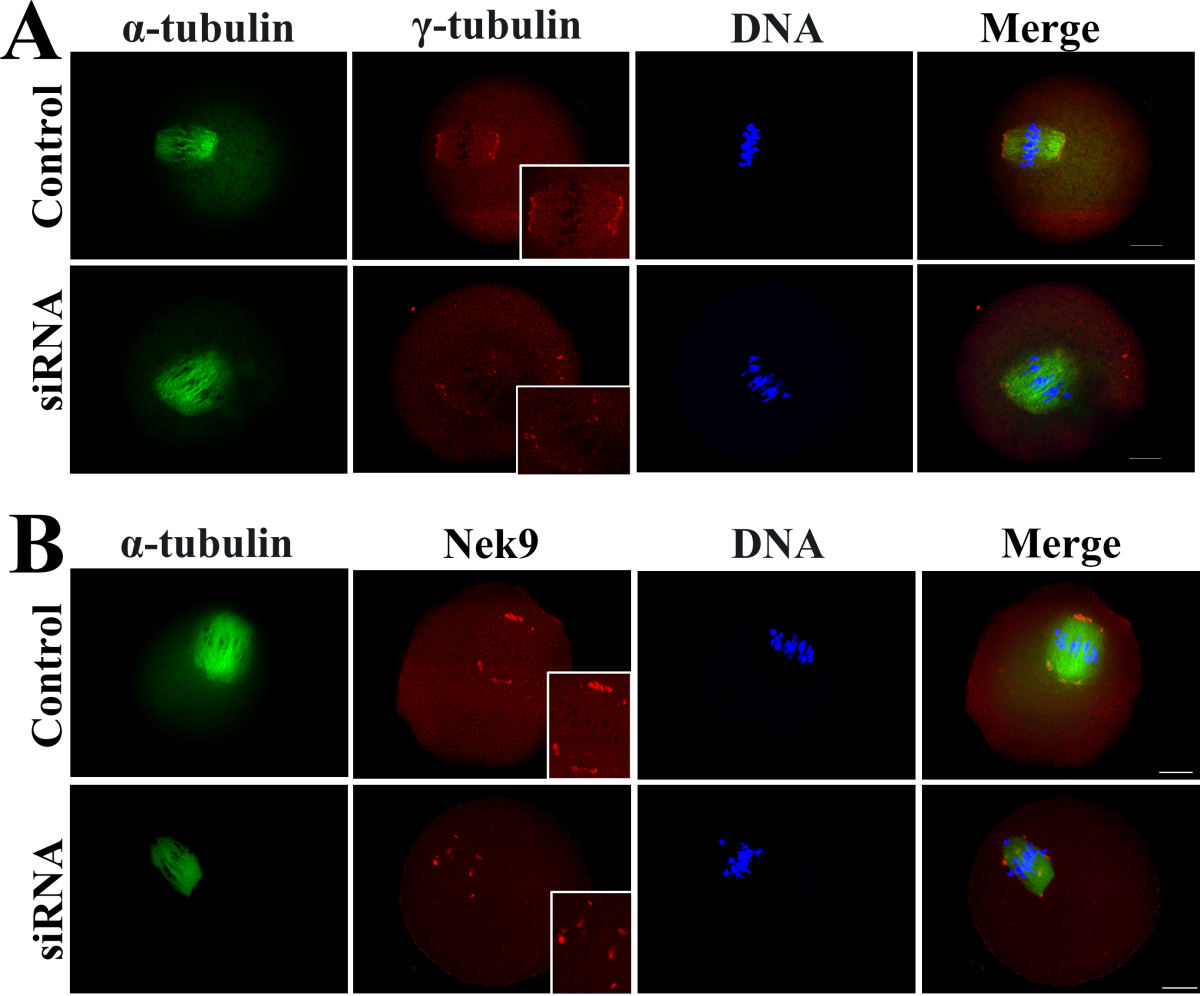
Dissociation of γ-tubulin and Nek9 from spindle poles in Axin1-knockdown oocytes at the MI stage. (A) Oocytes microinjected with Axin1 or control siRNAs were incubated in M2 for 8.5 h, and immunostained for α-tubulin (green), and γ-tubulin (red). DNA is stained with DAPI (blue). (B) In the Axin1 siRNA or control siRNA microinjection group, oocytes were cultured for 8.5 h, and then immunostained for α-tubulin (green) and Nek9 (red). DNA is stained with DAPI (blue). Scale bar = 20 μm.

The results shown above indicated that the Axin-1 protein might be involved in spindle organization. It is known that the MTOC-specific proteins γ-tubulin and Nek9 are important for microtubule nucleation and organization, leading to spindle formation during mouse oogenesis [23, 24], so we investigated the effect of Axin1 gene knockdown on the localizations of γ-tubulin and Nek9. We found that Axin-1 depletion affected the localization of γ-tubulin. As shown in **Fig 3A**, γ-tubulin was localized to the spindle poles in the control group. However, it failed to locate to the spindle poles and became scattered around the spindle in the Axin1 siRNA-injected group. Axin-1 depletion also affected the localization of Nek9. As shown in **Fig 3B**, Nek9 was localized to both spindle poles in the control group, whereas in the Axin1 siRNA-injected group it failed to concentrate at the spindle poles and instead was located on the spindle fibers.

### Knockdown of Axin1 gene expression decreased GVBD rate, PB1 extrusion and activated the spindle assembly checkpoint (SAC) protein BubR1

**Fig 4:**
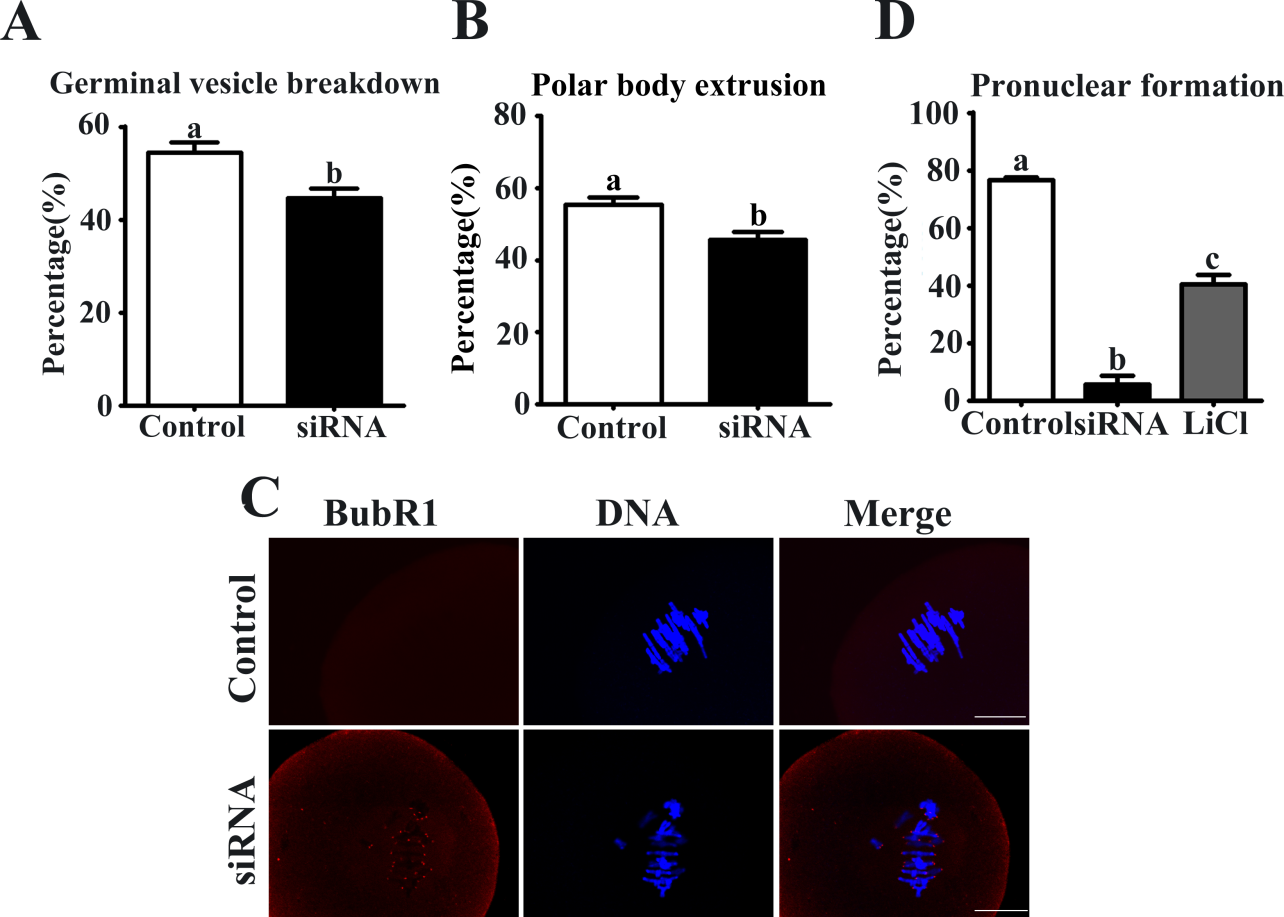
Axin-1 depletion decreased GVBD, PB1 extrusion and activated SAC. (A) The rate of GVBD in different group. (B) Percentages of first polar body (PB1) extrusion in the Axin1 siRNA microinjection and control group. (C) Detection of BubR1 (red) in oocytes in the control and Axin1 siRNA groups. DNA is stained with DAPI (blue). Scale bar = 20 μm. (D) The rates of pronuclear formation are shown in different groups. Data are presented as the mean ± SEM of three independent experiments. Different superscript letters indicate significant differences at P < 0.05.

After Axin1 or control siRNA microinjections, oocytes were cultured in fresh M2 medium for 12 h. The GVBD rate was reduced in the Axin1 siRNA-injected group compared with control (44.6 ± 2.0%, n = 198 vs. 54.4 ± 2.2%, n = 226) **(Fig 4A)**. The PB1 extrusion rate in the Axin1 siRNA-injected group (45.6 ± 2.2%, n = 198) was significantly lower than in the control group (55.3 ± 2.0%, n = 226; P < 0.05) (**Fig 4B**). Next, we analyzed the localization of BubR1 in oocytes from the Axin1 siRNA-injected group to explore activation of this SAC protein. Specific signals for BubR1 were detected in Axin1 gene knockdown oocytes, but the control oocytes showed no such signals (**Fig 4C**). Detection of BubR1 signal indicates activation of the SAC proteins.

### Knockdown of Axin1 gene expression decreased the pronuclear formation rate

After Axin1 or control siRNA microinjections, we activated the oocytes to determine the effects. As shown in **Fig 4D**, control oocytes exhibited a high rate of pronuclear formation (76.7 ± 0.9%, n = 80). However, a low rate was seen in the oocytes injected with Axin1 siRNA. Moreover, treatment with LiCl markedly increased the rate of pronuclear formation compared with the Axin1 siRNA-injected group (40.5 ± 3.3%, n = 67 vs. 5.8 ± 3.0%, n = 68).

## Discussion

We investigated the localization and potential functions of Axin-1 during meiotic maturation in mouse oocytes. Axin-1 was localized around spindles during this process. Downregulation of Axin1 gene expression by siRNA microinjection caused abnormal MTOC protein localization, disrupted the meiotic spindles, caused the chromosomes to misalign, led to activation of the SAC protein BubR1, and caused failure of PB1 extrusion. The addition of LiCl rescued the formation of normal spindles and pronuclei. These results suggest that the Axin-1 plays an important role in the structure of the spindle and the development of oocytes.

During mitosis, Axin-1 is localized to spindles and centrosomes [17, 18]. In our study, we found Axin-1 was located around the spindles during meiotic maturation. This localization of Axin-1 was analogous to those of other proteins, such as GM130 [25] and MEK1/2 [26], which are required for proper spindle formation in oocyte meiosis. Another study showed that Axin-1 was involved in microtubule nucleation in mitosis [17]. Thus, further study is necessary to determine exactly how Axin-1 is involved in spindle formation.

We found that Axin-1 might interact with centrosome-associated proteins to regulate spindle assembly in mouse oogenesis. We examined spindle morphology after depleting the level of the Axin-1 protein by siRNA microinjection. The Axin-1-depleted oocytes exhibited various types of morphologically defective spindles. Microtubules are primarily nucleated by centrosomes or MTOCs localized at the spindle poles [27]. γ-tubulin is a major component of the pericentriolar material of MTOCs. Nek9 colocalizes with γ-tubulin at MTOCs during meiosis and acts in regulating spindle organization [24]. Here we found that γ-tubulin and Nek9 became detached from the spindle poles in oocytes treated with siRNA for the Axin1 gene. This suggests that the Axin-1 protein might participate in regulating spindle organization by affecting integral and associated MTOC proteins.

The meiotic spindle is a vital structure for the events following fertilization such as the completion of meiosis, pronuclear migration, and formation of the first mitotic spindle [28]. We found a low rate of pronuclear formation in the Axin1 siRNA-injected group. Moreover, the culture medium supplemented with LiCl—a Wnt pathway activator [29–31]—improved the pronuclear formation rate. We also found fewer disorganized spindles in the siRNA injected with LiCl group. Clearly, Axin-1 is important for oocyte meiosis.

Next, we found that Axin-1 depletion led to the failure of PB1 extrusion. However, abnormal spindle assembly caused by the loss of function of key proteins such as BRCA1 might not always affect PB1 extrusion [32]. In some other studies, PB1 extrusion was decreased when key proteins were disrupted. For example, depletion of Nek9 was shown to activate the SAC and cause arrest at the pro-MI/MI stage [24]. As Axin-1 depletion decreased the PB1 extrusion rate, such meiotic arrest might be related to SAC functions. During mammalian mitosis and meiosis, the SAC system acts to prevent premature progression to anaphase until all chromosomes are correctly attached to the spindle. The SAC proteins include mitotic Mad1, Mad2, BubR1, and Mps1 [4, 33–36]. Here, we detected specific signals for BubR1 in the Axin1 siRNA group, while in the control group we did not detect any such signal. This result confirmed that Axin-1 depletion activated the SAC protein BubR1 at MI, and then disrupted PB1 extrusion.

## Conclusions

Our data strongly suggest that Axin-1 regulates spindle assembly, chromosome alignment, and the metaphase–anaphase transition during meiotic maturation in mouse oocytes.

## Supporting Information

S1 Original Data (DOC)

## References

[1] Niikura Y, Niikura T, Tilly JL. Aged mouse ovaries possess rare premeiotic germ cells that can generate oocytes following transplantation into a young host environment. Aging. 2009;1:971–8. 20157580 10.18632/aging.100105 PMC2815754

[2] Niikura Y, Niikura T, Wang N, Satirapod C, Tilly JL. Systemic signals in aged males exert potent rejuvenating effects on the ovarian follicle reserve in mammalian females. Aging. 2010;2:999–1003. 21212462 10.18632/aging.100255 PMC3034188

[3] Zhu XL, Qi ST, Liu J, Chen L, Zhang C, Yang SW, et al Synaptotagmin1 is required for spindle stability and metaphase-to-anaphase transition in mouse oocytes. Cell Cycle. 2012;11:818–26. 10.4161/cc.11.4.19329 22313732

[4] Homer H, Gui L, Carroll J. A spindle assembly checkpoint protein functions in prophase I arrest and prometaphase progression. Science. 2009;326:991–4. 10.1126/science.1175326 19965510 PMC3428834

[5] Manandhar G, Schatten H, Sutovsky P. Centrosome reduction during gametogenesis and its significance. Biology of reproduction. 2005;72:2–13. 15385423 10.1095/biolreprod.104.031245

[6] Schuh M, Ellenberg J. Self-organization of MTOCs replaces centrosome function during acentrosomal spindle assembly in live mouse oocytes. Cell. 2007;130:484–98. 17693257 10.1016/j.cell.2007.06.025

[7] Gunawardane RN, Lizarraga SB, Wiese C, Wilde A, Zheng YX. gamma-tubulin complexes and their role in microtubule nucleation. Centrosome in Cell Replication and Early Development. 2000;49:55–73. 10.1016/s0070-2153(99)49004-0 11005014

[8] Yang S-W, Gao C, Chen L, Song Y-L, Zhu J-L, Qi S-T, et al Nek9 regulates spindle organization and cell cycle progression during mouse oocyte meiosis and its location in early embryo mitosis. Cell Cycle. 2014;11:4366–77. 10.4161/cc.22690 PMC3552919 23159858

[9] Salahshor S, Woodgett JR. The links between axin and carcinogenesis. Journal of clinical pathology. 2005;58:225–36. 15735151 10.1136/jcp.2003.009506 PMC1770611

[10] Kaplan DD, Meigs TE, Kelly P, Casey PJ. Identification of a role for beta-catenin in the establishment of a bipolar mitotic spindle. The Journal of biological chemistry. 2004;279:10829–32. 14744872 10.1074/jbc.C400035200

[11] Fodde R, Kuipers J, Rosenberg C, Smits R, Kielman M, Gaspar C, et al Mutations in the APC tumour suppressor gene cause chromosomal instability. Nature cell biology. 2001;3:433–8. 11283620 10.1038/35070129

[12] Green RA, Wollman R, Kaplan KB. APC and EB1 function together in mitosis to regulate spindle dynamics and chromosome alignment. Molecular biology of the cell. 2005;16:4609–22. 16030254 10.1091/mbc.E05-03-0259 PMC1237068

[13] Wakefield JG, Stephens DJ, Tavare JM. A role for glycogen synthase kinase-3 in mitotic spindle dynamics and chromosome alignment. Journal of cell science. 2003;116:637–46. 12538764 10.1242/jcs.00273

[14] Zeng L, Fagotto F, Zhang T, Hsu W, Vasicek TJ, Perry WL 3rd, et al The mouse Fused locus encodes Axin, an inhibitor of the Wnt signaling pathway that regulates embryonic axis formation. Cell. 1997;90:181–92. 9230313 10.1016/s0092-8674(00)80324-4

[15] Zhang Y, Neo SY, Wang X, Han J, Lin SC. Axin forms a complex with MEKK1 and activates c-Jun NH(2)-terminal kinase/stress-activated protein kinase through domains distinct from Wnt signaling. The Journal of biological chemistry. 1999;274:35247–54. 10575011 10.1074/jbc.274.49.35247

[16] Li Q, Lin S, Wang X, Lian G, Lu Z, Guo H, et al Axin determines cell fate by controlling the p53 activation threshold after DNA damage. Nature cell biology. 2009;11:1128–34. 19731416 10.1038/ncb1927

[17] Fumoto K, Kadono M, Izumi N, Kikuchi A. Axin localizes to the centrosome and is involved in microtubule nucleation. EMBO reports. 2009;10:606–13. 10.1038/embor.2009.45 19390532 PMC2711835

[18] Kim SM, Choi EJ, Song KJ, Kim S, Seo E, Jho EH, et al Axin localizes to mitotic spindles and centrosomes in mitotic cells. Experimental cell research. 2009;315:943–54. 10.1016/j.yexcr.2009.01.013 19331826

[19] Ruan K, Ye F, Li C, Liou YC, Lin SC, Lin SY. PLK1 interacts and phosphorylates Axin that is essential for proper centrosome formation. PloS one. 2012;7:e49184 10.1371/journal.pone.0049184 23155463 PMC3498349

[20] Huang X, Ding L, Pan R, Ma PF, Cheng PP, Zhang CH, et al WHAMM is required for meiotic spindle migration and asymmetric cytokinesis in mouse oocytes. Histochemistry and cell biology. 2013;139:525–34. 10.1007/s00418-012-1051-z 23160625

[21] Liu Y, He XQ, Huang X, Ding L, Xu L, Shen YT, et al Resveratrol protects mouse oocytes from methylglyoxal-induced oxidative damage. PloS one. 2013;8:e77960 10.1371/journal.pone.0077960 24194906 PMC3806792

[22] Shen YT, Song YQ, He XQ, Zhang F, Huang X, Liu Y, et al Triphenyltin chloride induces spindle microtubule depolymerisation and inhibits meiotic maturation in mouse oocytes. Reproduction, fertility, and development. 2013. 10.1071/RD12332 23981671

[23] Sonn S, Oh GT, Rhee K. Nek2 and its substrate, centrobin/Nip2, are required for proper meiotic spindle formation of the mouse oocytes. Zygote (Cambridge, England). 2011;19:15–20. 10.1017/S0967199410000183 20569513

[24] Yang SW, Gao C, Chen L, Song YL, Zhu JL, Qi ST, et al Nek9 regulates spindle organization and cell cycle progression during mouse oocyte meiosis and its location in early embryo mitosis. Cell Cycle. 2012;11:4366–77. 10.4161/cc.22690 23159858 PMC3552919

[25] Zhang CH, Wang ZB, Quan S, Huang X, Tong JS, Ma JY, et al GM130, a cis-Golgi protein, regulates meiotic spindle assembly and asymmetric division in mouse oocyte. Cell Cycle. 2011;10:1861–70. 21552007 10.4161/cc.10.11.15797

[26] Sun SC, Xiong B, Lu SS, Sun QY. MEK1/2 is a critical regulator of microtubule assembly and spindle organization during rat oocyte meiotic maturation. Molecular Reproduction and Development. 2008;75:1542–8. 10.1002/mrd.20891 18270979

[27] Wiese C, Zheng Y. Microtubule nucleation: gamma-tubulin and beyond. Journal of cell science. 2006;119:4143–53. 17038541 10.1242/jcs.03226

[28] Schatten G, Simerly C, Schatten H. Microtubule configurations during fertilization, mitosis, and early development in the mouse and the requirement for egg microtubule-mediated motility during mammalian fertilization. Proceedings of the National Academy of Sciences of the United States of America. 1985;82:4152–6. 3889922 10.1073/pnas.82.12.4152 PMC397953

[29] Shan T, Zhou C, Yang R, Yan F, Zhang P, Fu Y, et al Lithium chloride promotes the odontoblast differentiation of hair follicle neural crest cells by activating Wnt/beta-catenin signaling. Cell biology international. 2015;39:35–43. 10.1002/cbin.10340 25044369

[30] Yang Y, Yang J, Liu R, Li H, Luo X, Yang G. Accumulation of beta-catenin by lithium chloride in porcine myoblast cultures accelerates cell differentiation. Molecular biology reports. 2011;38:2043–9. 10.1007/s11033-010-0328-3 20857211

[31] Zhang N, Dai YL, Huang LF, Liu WL. [Therapeutic effect of lithium chloride combined with cyclosporine A on mouse model with aplastic anemia]. Zhongguo shi yan xue ye xue za zhi / Zhongguo bing li sheng li xue hui = Journal of experimental hematology / Chinese Association of Pathophysiology. 2012;20:654–7. 22739176

[32] Xiong B, Li S, Ai JS, Yin S, OuYang YC, Sun SC, et al BRCA1 is required for meiotic spindle assembly and spindle assembly checkpoint activation in mouse oocytes. Biology of Reproduction. 2008;79:718–26. 10.1095/biolreprod.108.069641 18596218

[33] Althoff F, Karess RE, Lehner CF. Spindle checkpoint-independent inhibition of mitotic chromosome segregation by Drosophila Mps1. Molecular biology of the cell. 2012;23:2275–91. 10.1091/mbc.E12-02-0117 22553353 PMC3374747

[34] Hached K, Xie SZ, Buffin E, Cladiere D, Rachez C, Sacras M, et al Mps1 at kinetochores is essential for female mouse meiosis I. Development (Cambridge, England). 2011;138:2261–71. 10.1242/dev.061317 21558374

[35] Homer HA. Mad2 and spindle assembly checkpoint function during meiosis I in mammalian oocytes. Histology and histopathology. 2006;21:873–86. 16691540 10.14670/HH-21.873

[36] Wei L, Liang XW, Zhang QH, Li M, Yuan J, Li S, et al BubR1 is a spindle assembly checkpoint protein regulating meiotic cell cycle progression of mouse oocyte. Cell Cycle. 2010;9:1112–21. 20237433 10.4161/cc.9.6.10957

