# Supplementary material for: Axin-1 Regulates Meiotic Spindle Organization in Mouse Oocytes
Source: PLoS One. 2016 Jun 10;11(6):e0157197. doi: 10.1371/journal.pone.0157197 (PMC4902301; doi:10.1371/journal.pone.0157197)
Supplement: S1 Original Data — (DOC) [file pone.0157197.s001.doc]

1. Real time PCR

Ct (Control) 2.953236262 3.427637736 3.190436999

Ct (siRNA) 5.578584035 5.307349523 5.933982213

2. Chromosome alignment

Normal Abnormal

Group 1

Control 20 8

siRNA 16 19

Group 2

Control 21 4

siRNA 19 6

Group 3

Control 29 5

siRNA 12 20

3. Spindle formation

Normal Abnormal

Group 1

Control 21 7

siRNA 7 28

Group 2

Control 17 8

siRNA 12 13

Group 3

Control 19 15

siRNA 8 24

4. PB formation

GVBD PB

Group 1

Control 34 42

siRNA 35 27

Group 2

Control 30 43

siRNA 38 38

Group 3

Control 37 40

siRNA 34 26

Captions：

**1. Ct values in different groups.**

**2. The statistics of oocytes with abnormal chromosome alignment in different groups.** After siRNA injection, oocytes were fixed and labeled with DAPI and were analyzed with confocal microscopy.

**3.** **The statistics of oocytes with abnormal Spindle formation in different groups.** After siRNA injection, oocytes were fixed and labeled with FITC-a-tubulin and were analyzed with confocal microscopy.

**4.** **The statistics of oocytes with polar body formation in different groups.** After siRNA injection, oocytes were cultured 12 h. Afterwards, polar body extrusion were estimates using the equation:

**PBE% =** (Number of oocytes with polar body extrusion) / (Number of oocytes with germinal vesicle breakdown)
